# Female breast cancer in Vietnam: a comparison across Asian specific regions

**DOI:** 10.7497/j.issn.2095-3941.2015.0034

**Published:** 2015-09

**Authors:** Phuong Dung (Yun) Trieu, Claudia Mello-Thoms, Patrick C. Brennan

**Affiliations:** Faculty of Health Sciences, University of Sydney, New South Wales 2141, Australia

**Keywords:** Breast cancer, Vietnam, Southeast Asia (SEA), Australia

## Abstract

Breast cancer is one of the most commonly diagnosed malignancies and the leading cause of cancer death of women over the world. A large number of females with breast cancer in Vietnam and other Southeast Asian (SEA) countries present at an early age with more aggressive tumors compared with women in Australia. Despite experiencing a low incidence rate, the increasing incidence rate among SEA countries exceeds that of the Westernized world. Changes in reproductive factors, environmental exposures, and lifestyle are the possible causes of this trend. However, limited evidence shows that these factors are associated with breast cancer in the Vietnamese population. Breast cancer incidence rates within Vietnam are not uniform and appear to be dependent on geographic location. Findings from this review have important implications for breast cancer control and treatment in Vietnam. A good understanding of the morphology of the breast and the type and nature of breast cancers presenting in Vietnam is required to facilitate the introduction of an effective national breast screening program.

## Introduction

Breast cancer is the most frequent neoplasm occurring in women globally. Approximately 1.7 million women worldwide were diagnosed with breast cancer and over 522,000 women died from this disease in 2012^1^. Although it is thought to be a disorder primarily of the developed world due to the high incidence rate, 50% of new breast cancer cases occurred in less developed countries in 2007^2^, and this number reached 53% in 2012^3^. Breast cancer is predicted to continue to increase in less developed countries over the next decade and become the leading cause of cancer-related deaths among women throughout the world^1^.

Vietnam is a developing country located in Southeast Asia (SEA) consisting of 64 cities and a population of 90 million people. Vietnam is a low-income nation with an annual wage per capita of US$3,200 and voluntary social health insurance of approximately US$20.00 per annum, which is paid by individuals and their households. Despite the inadequate public health conditions, Vietnam has a significantly low age-standardized incidence rate for breast cancer at 23 per 100,000 women^3^ compared with approximately 120 per 100,000 women reported in developed Western countries, such as Australia and the United States. Nonetheless, in the developed world, this rate has been stable or showing signs of decrease; the rate has increased in Vietnam by a factor greater than two over the last two decades, making this disease becoming the most frequently diagnosed cancer among Vietnamese women^3^. Given the scarcity of published data across Asian countries, typical of developing regions, most of the incidence rates used in this review were sourced from the International Agency for Research on Cancer (IARC) published in 2012.

The aim of this paper is to review the status of female breast cancer in Vietnam and other SEA countries and compare reported values with data available in Australia (a typical Westernized country). Such comparative exercises are important because an understanding of incidence figures, studied alongside culturally specific information on genetic background, socio-economic profile, lifestyle, behavior, and health beliefs, may throw some light on the causal agents and risk factors associated with breast cancer. Most of our current knowledge on breast cancer has been generated from studies conducted in Western populations. To maximize a global and effective regional-specific response to this disease, changes in the burden of breast cancer should be monitored over time in other populations. This approach allows some degree of benchmarking between countries and facilitates the allocation of resources to support the effective preventative, diagnostic, and curative strategies to improve breast cancer management in developing countries such as Vietnam.

## Breast cancer incidence

### Across SEA

**Figure 1:**
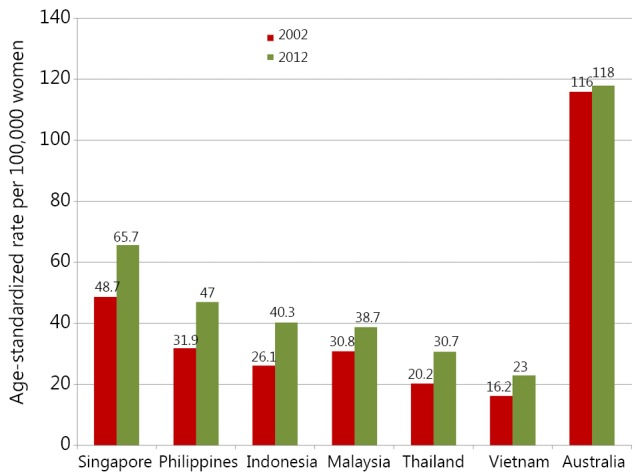
Comparison of breast cancer incidence rates in Vietnam and other SEA countries between 2002 and 2012 (Sources: GLOBOCAN^3^ and Australia Department of Health^7^). SEA, Southeast Asia.

According to the IARC 2012, breast cancer was one of the two most common cancers among women in SEA, making up 22.4% of all cancers with an age-standardized rate (ASR) of 27.8 per 100,000. Within specific countries in the region, the values of incidence can substantially exceed those reported in Vietnam (**Table 1**), which currently is estimated to have the lowest breast cancer incidence rate in SEA (23 per 100,000). The highest ASR is found in Singapore, with 65.7 per 100,000 women being affected. Rapid economic growth and low unemployment rates have converted Singapore from a developing country to a developed country within four decades, with rising standards of living and advanced healthcare facilities^4^. Nonetheless, throughout that period, the incidence rate of newly diagnosed breast cancers in Singapore females per 100,000 increased threefold from 21.5 in 1971-1975 to 60.7 in 2006-2010 and to 65.7 in 2012, a rate now 3 times higher than that of Vietnam^5^^,^^6^. The Philippines also has a relatively high incidence with 1 in every 13 Filipino women expected to develop breast cancer in her lifetime, and the ASR being 47 per 100,000 women in 2012, a 25% increase compared with 10 years earlier. Indonesia and Malaysia are next in terms of incidence ranking at 40.3 and 38.7 per 100,000, whereas Thailand overtook Vietnam in 2012 with 29.3 women developing breast cancer in every 100,000. Similar to other SEA countries, the increasing rate of breast cancer is also a trend in Malaysia, Indonesia, and Thailand, which have witnessed a growth of 25.6%, 54.4%, and 76.5%, respectively, between 2002 and 2012 (**Figure 1**). Although the rate of breast cancer is rising noticeably in SEA countries, this rate is still much lower than that of Westernized countries.

**Table 1:** Breast cancer incidence rate in countries in 2012 (Sources: GLOBOCAN and Australia Department of Health, 2012)

Countries	Data quality*	Population (in millions)	No. of breast cancers	Incidence rates (%)
Singapore	A	5.3	2,524	65.7
Philippines	B	103.8	18,327	47.0
Indonesia	F	248.2	48,998	40.3
Malaysia	C	29.2	5,410	38.7
Thailand	B	67.1	13,653	29.3
Vietnam	E	89.8	11,067	23.0
Australia	A	22	14,710	118

*Note: A, high quality national data or high quality regional (coverage greater than 50%); B, high quality regional (coverage between 10% and 50%); C, high quality regional (coverage lower than 10%); D, national data (rates); E, regional data (rates); F, frequency data.

As a benchmark, the above data should be compared with those from more developed regions. Along with the United States, Australia has been considered as one of the top countries threatened by breast cancer. Studies show that one in every eight Australian women will develop breast cancer in their lifetime^7^. The ASR of female breast cancer in Australia in 2012 (118 per 100,000) is approximately twice as high as the rate in Singapore and almost six times of Vietnam. Despite being among the top countries threatened by breast cancer, the incidence rate in Australia remained almost stable in the last three decades^7^.

The reasons for these temporal escalations in incidence rates in SEA should be explored. Changes in living standards and lifestyles have affected age at first pregnancy and the number of children being born. Traditionally, women in SEA would marry and bear children in their early 20s and breastfeed their babies for long periods, all of which were protective agents against breast cancer^8^^,^^9^. However, the increasing occupational and tertiary educational opportunities for women influences their behavior, with many postponing marriages, having fewer children, and more remaining single^10^. The average age at first marriage of SEA women increased from 21.6 in 1970 to 26.2 in 2010, and the fertility rate per woman decreased significantly from 4.9 to 2.0 in the same period of time (**Table 2**)^11^^,^^12^. In addition, considerable evidence exists that SEA women are adopting a Western lifestyle, and the percentage of obese women increased from 2.3% in 1980 to 8.6% in 2008^13^; in the Philippines, a plethora of fast food outlets have become evident, and subsequently, Filipinos have been shown to have adopted eating and drinking habits which resemble that of US citizens and which have been associated with higher breast cancer risk^14^. These examples suggest that lifestyles of SEA women are resembling those reported for Westernized countries (i.e., over-eating, over-drinking, and less physical exercise), in combination with having fewer children at later stages becoming more evident^15^^-^^18^.

**Table 2:** Trends in age at first marriage and fertility rate of SEA women (Source: WorldBank, 2012)

Countries	1970			1990			2010	
	Marriage age	Fertility rate (%)		Marriage age	Fertility rate (%)		Marriage age	Fertility rate (%)
Indonesia	19.3	5.5		21.6	3.5		24	2.4
Malaysia	22.1	4.7		24.6	4		26.9	2.1
Philippines	22.8	5.2		23.8	4.3		26.7	3.2
Singapore	24.2	2.7		27	1.7		29.3	1.2
Thailand	22	5		23.5	2.6		25.8	1.4
Vietnam	19.5	6.4		20.6	4.2		24.5	1.8
Average	21.7	4.9		23.5	3.4		26.2	2.0

Globally, one in three women diagnosed with breast cancer was estimated to be under 50 at the time of diagnosis compared with 42% in the Asia-Pacific region and 47% within SEA specifically^19^. The incidence peak age across SEA countries ranges from 44 to 69 years old (**Table 3**), with the Philippines and Indonesia recording the lowest mean ages of 44 and 47 years old^20^^,^^22^, whereas Thailand and Malaysia having some of the highest recorded ages (i.e., 50 to 54 years old)^25^^,^^28^. Among SEA countries, only Singapore recorded a similar age pattern to that of Australia, where over 60% of breast cancer patients were older than 50 years old^6^ with diagnosis occurring most frequently between the ages of 50 and 69 years.

**Table 3:** Breast cancer age peak and stages in the SEA countries and Australia

Countries	Period	Population	Age peak	Stages (%)				
				0 (*in situ*)	1	2	3	4
Philippines								
Matsuda *et al*. (2002)^20^	1997-2000	294	44		3	32	52	1
Ngelangel and Wang (2002)^21^	1988-1991	283	47		2	37	55	6
Indonesia								
Ng *et al*. (2011)^22^	2010	637	47	6	27	34	25	8
Aryandono *et al*. (2006)^23^	1993-2003	223	49		15	4	37	18
Thailand								
Kotepui and Chupeerach (2013)^24^	2002-2011	7,711	50-54	1	29.7	13.2	36.4	8.9
Malaysia								
Pathy *et al*. (2011)^25^	1993-2007	3,320	50	2.9	21.6	42.4	22.3	10.8
Ng *et al*. (2011)^22^	2010	477	52	0	27	34	25	8
Singapore								
Pathy *et al*. (2011)^25^	1993-2007	2,141	50	1	24.7	42.9	14.4	7.9
Vietnam								
Nguyen *et al*. (2009)^26^	2001-2007	1,584	50	4	10.7	61.2	19.4	8.23
Australia								
AIHW (2010)^27^	2008	13,567	65-69	72		28		

Women with breast cancer in SEA appear not only in early age but also with late stage disease and are rarely diagnosed with “pre-invasive” cancer, such as ductal carcinoma *in situ* or lobular carcinoma *in situ*, in contrast to Australia where 72% of breast cancer patients present with non-invasive tumors^27^. Over 50% of breast cancer patients in SEA were diagnosed with locally advanced stage III or distantly advanced stage IV^29^, with Singapore being, again, the exception where 69% of breast cancer cases were diagnosed in early stages^6^. In the Philippines, 53% of breast cancers appear in stages III and IV and only 2%-3% in stage I^20^^,^^21^, whereas in Indonesia and Thailand, the proportion of women with late stage breast cancer ranges from 45% to 55%. In Indonesia, 55% of cases appear as invasive ductal carcinoma^23^, similar to Thailand with the rate between 76% and 91% across nine registries^24^.

### Vietnam

#### Overview

Breast cancer is currently the most common cancer among Vietnamese women (**Table 4**)^30^. The rate of women diagnosed with breast cancer is generally relatively low in Vietnam, but the recently reported increases demand attention. In 2012, approximately 11,060 cases of female breast cancer were diagnosed, with 64.7% of the cases below age 50. These data positioned breast cancer as the leading cancer among Vietnamese women or fifth in all female cancer cases^31^. This situation changed from 1993-1998 when the cervix and uterus were the most affected organs at a rate of 17.8/100,000 women, which was just ahead of breast cancer incidence at 17.3/100,000^31^. The relatively low incidence rate of breast cancer in Vietnam compared with other countries is traditionally associated with high level of fertility (first pregnancy at early age; multiple births) and prolonged breast feeding of infants. However, the recent fall of fertility rate in Vietnam, which is partly due to the two-child policy that took effect since 1988, has led to a dramatic change in the average number of children produced by Vietnamese women, falling from 7.28 in 1962 to 1.77 in 2012^12^. In addition, the rapid increase in the number of obese women in Vietnam from 5% in 1980 to 12% in 2013^32^ may have contributed to the recent growth in breast cancer incidence. Moreover, improved health services may have been a factor. In 1995, Vietnam spent US$14 per capita on health expenditure and increased to US$102 per capita in 2012^33^, resulting in three times more physicians^34^ and a vastly increased availability of diagnostic tests.

**Table 4:** Top 10 common cancers in Vietnamese women and difference between Ha Noi and Ho Chi Minh City (Source: Vietnam Department of Health, 2008)

No	Ha Noi			Ho Chi Minh City	
	Site	ASR		Site	ASR
1	Breast	29.7		Breast	19.4
2	Stomach	15		Cervix	16.5
3	Lung	10.5		Lung	12.4
4	Colorectum	10.1		Colorectum	9
5	Cervix	9.5		Liver	6
6	Thyroid	5.6		Stomach	5.5
7	Ovary	4.7		Ovary	3.8
8	Liver	4.5		Thyroid	3.8
9	Non-Hodgkin lymphoma	4		Non-Hodgkin lymphoma	3.2
10	Leukemia	3.4		Skin	2.6

#### Age of women and stage of cancer

**Figure 2:**
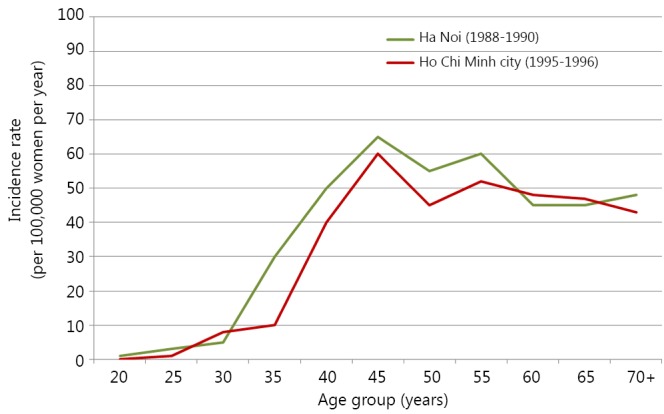
Distribution of breast cancer incidence rates among age groups in Ha Noi and Ho Chi Minh city^26^^,^^36^.

The most common age group of women with breast cancer in Vietnam is 45 to 55 years old. The age incidence relationship in Ha Noi and Ho Chi Minh City (HCMC) in the north and the south of Vietnam, respectively, is almost identical with the risk increasing at ages 45-55 and then flattens off or even decline after menopause^35^^,^^36^ (**Figure 2**). Unlike in Western countries where women typically have early stages of the disease, a large number of breast cancers in Vietnam occur at a later stage of development, making the treatment more difficult. The percentage of breast cancer patients in Vietnam with stage 0-I and II is 14.7% and 61.2%, respectively, whereas that of the distantly advanced stages (III,IV) is 27.6%^26^. These figures are in contrast to that in the United States, where 58.6% of cancers were diagnosed at stage I^37^ and 72% of cases in Australia resulted from non-invasive cancers^27^. The advanced stage of the disease in Vietnam is most likely due to poor public awareness, lack of breast screening programs, and low numbers of general practitioners at the grass root levels of the health care system^31^.

#### Geographic variations

The incidence rates of breast cancer within Vietnam are not uniform and appear to be dependent on geographic location. Therefore, we should explore why specific variations occur, particularly in a country like Vietnam where large geographic-dependent ethnic variations do not obviously exist; however, causal agents are not immediately identifiable. Ha Noi and HCMC are the largest cities and leading centers in economy, education, and health care, have similar population sizes of 6.5 and 7.4 million, and are ethnically similar with 98.7% and 94% of the total population being Vietnamese, respectively. Both cities appear to have similar modern medical facilities and specialized hospitals, each being the major hubs of cancer diagnosis and treatment in the north and the south of Vietnam. Nevertheless, Ha Noi has a higher incidence rate (26.7 per 100,000), which is more than twice higher than the rate in HCMC (12.2 per 100,000)^35^^,^^36^. Although this difference had narrowed down in 2001-2004, the rate in the capital city in the north of Vietnam (29.7) still exceeded^38^ that of the largest city in the south (19.4)^30^. Reports from the Cancer Registries in Ha Noi and HCMC have not only highlighted this difference in breast cancer incidence but also noted that there was four times higher rate of cervical cancer in the southern city (26 per 100,000) compared with the northern city (6.3 per 100,000). This finding has been linked to high rates of human papilloma virus (HPV) infections during the Second Indochinese War in South Vietnam, where a woman whose husband had served in the army experienced a 160% increased risk of cervical cancer compared with a woman whose husband had not served in the military. Concomitantly, in northern Vietnam, a 290% increase of cervical cancer was evident particularly among women whose husbands were in the military and stationed in South Vietnam during the war relative to women with civilian husbands^38^.

Although some evidence is available accounting for the variations in cervical cancer incidence, associations linked to breast cancer variations are much less developed. Nonetheless, the following possibilities have been proposed and dismissed: toxic chemicals used during the war such as dioxin are excluded because the south and not the north population received the heaviest exposure^39^; chlorinated insecticide used widely in rural areas in the north of Vietnam for control of malaria is excluded due to the lack of correlation between the blood levels of p,p’-DDT/p,p’-DDE (ingredients of insecticide) and breast cancer risk found among northern residents^40^.

Although differences are found in terms of cultural customs and climate between Ha Noi and HCMC, the lifestyles in the two cities are relatively similar, and we must determine lifestyle agents that may account for regional variations in breast cancer incidence. The development in the agricultural sectors and economic growth in the last two decades transformed Vietnam from a country being highly dependent on food aid to a food exporter country, from a country of famine to one with a surplus of food, and from a country of staple-based diets to one with more balanced and nutritious diets. As a result, the prevalence of Vietnamese women aged 20-49 years with a body mass index (BMI) less than 18.5 (underweight) was reduced from 33.1% in 1990 to 26.3% in 2000^41^. Nonetheless, even with these overall changes, some differences were found in the health indicators of women in the north and south of Vietnam. Although more women have inadequate daily physical activities [MET (metabolic equivalent) hours per week] in the south (41.2%) than in the north (13.7%), possible agents for breast cancer risk, such as obesity, smoking, and alcohol consumption, are more common with women in Ha Noi than in HCMC, with rates of obesity and smoking in women in Ha Noi (17.4%, 4.3%) being almost triple to that of HCMC (5.9%, 1.6%); the proportion of women with excessive alcohol consumption (more than 2 standard units of drink per day) in the north (1.1%) is twice that of the south (0.4%)^42^^,^^43^. With the exception of physical activity, these data may explain the higher rate of breast cancer in the north.

#### Other risk factors related to breast cancer in Vietnam

Researchers have investigated other breast cancer risk factors in Vietnam. In a case-control study conducted in 6 hospitals in Vietnam and 1 in China, which involved 682 patients with 649 controls (93.5% living in Vietnam and 6.5% living in China), authors found that 56% Vietnamese women had their first baby before the age of 25. However, premenopausal women who had their first full-term pregnancy after the age of 25 had a 1.5 times risk of developing breast cancer compared with women who had their first full-term pregnancy before the age of 25^44^. However, unlike data available elsewhere for other countries^8^^,^^15^^,^^45^^,^^46^, BMI, age, menarche, and total months of lactation were not significantly associated with breast cancer risk in Vietnam^44^. In another study, researchers did not also find any relationship between family history, BRCA mutations, and breast cancer in 298 patients in Ha Noi^47^. These clear differences in risk factors between Vietnamese and Caucasian women demand specific attention and cautions against generalizations about breast cancer risks.

One potential agent associated with breast cancer risk that has not been explored in Vietnam is mammographic breast density (MBD). MBD is a radiological parameter that represents the amount of fibro-glandular tissue within the breast. In North America, women with dense breasts have been shown to have a four- to sevenfold increased risk of developing breast cancer^48^, and this risk extends for at least 8-10 years after breast density assessment^49^. Researchers also found that the percentage of Australian women with dense breasts is 33.4%, and the chance of Australian women with the highest category of dense tissue to develop breast cancer is four times higher than the women in the lowest density category^50^. Closer to SEA, Singapore women with more than 75% breast density have almost five times the risk of breast cancer compared with those having a density of 10% or less^51^. No breast density data of Vietnam are available at current time; thus, breast cancer risk factors cannot be fully assessed; regional differences in cancer incidence cannot be explained; and given that cancer detection is dependent on mammographic density, imaging strategies cannot be optimized.

## Breast screening programs

As previously noted, a large proportion of breast cancer patients in Vietnam and other SEA countries are diagnosed with advanced stages of the disease, and up to 25% have distant metastases at initial presentation^25^. Therefore, demands are made for the introduction of population-based mass screening mammography in Asian women over recent years. Mammography with reasonably high sensitivity (60%-80%) and specificity (73%-95%) has been considered to be a reliable modality in detecting early breast lesions through abnormal signs, such as masses, calcifications, bilateral asymmetry, and distortion^52^^,^^53^. Following the introduction of mammography screening programs in developed countries, improvements in breast cancer detection and decreases in mortality rates have been made. In a case-control study of patients participating in Breast Screen Australia between 2002 and 2005, authors found a 41% decrease in death due to breast cancer in participating women^54^ compared with those women who did not attend. Although breast screening programs are proved to be effective in increasing the early detection of breast cancer in Westernized countries, little evidence is available that breast cancer screening through mammography would also be effective in the SEA setting. Approximately 50% of SEA women are diagnosed with breast cancer before the age of 50 and Asian women generally have small-volume breasts and relatively dense parenchymal breast tissue, which may obscure detection of early and small breast tumors^55^. In addition, financial and logistic barriers are evident in a number of countries^56^. Singapore is the first country that underwent a population-based screening after a trial program in which 166,600 women aged 50-64 years old were randomly allocated to screening by mammography or non-screening observation; the screening demonstrated an increase in breast cancer detection from 1.3-4.6 per 1,000 women in the former group^57^. Some other SEA countries have established guidelines for the frequency of mammography in detecting breast cancer, but national screening programs are still not widely available. In Vietnam, despite increased health-care and public awareness, a breast cancer national control program as recommended by the WHO has not yet been established. This recommendation is at least partly due to the breast cancer incidence being relatively low and to the lack of research examining the cost-effectiveness of introducing a nationwide screening policy. It is hard to visualize changes to this program in the near future unless the specific benefits and relevance of radiologic modalities to the breast morphology of Vietnamese women is established.

## Conclusion

This review has shown that a large number of females in Vietnam and other SEA countries are diagnosed with breast cancer at an early age with more aggressive tumors. Despite experiencing a low incidence, the increasing incidence rate among SEA countries exceeds that of the Western world, with Australia as an example. Changes in reproductive factors, environmental exposures, and lifestyle have all been proposed to explain this trend. However, the evidence that these factors are associated with breast cancer in Vietnamese population is limited.

An effective breast screening program will most likely be beneficial to Vietnam, but until more is known around the morphology of the Vietnamese breast and the type of cancers being presented, the type and cost-effectiveness of such a program cannot be established. Nonetheless, given the current incidence trends, some forms of screening need to be established and, therefore, high quality data are required to inform the choice of radiologic modality, the frequency of examination, and the group of women to be prioritized.
